# CRISPRi-Manipulation of Genetic Code Expansion via RF1 for Reassignment of Amber Codon in Bacteria

**DOI:** 10.1038/srep20000

**Published:** 2016-01-28

**Authors:** Bo Zhang, Qi Yang, Jingxian Chen, Ling Wu, Tianzhuo Yao, Yiming Wu, Huan Xu, Lihe Zhang, Qing Xia, Demin Zhou

**Affiliations:** 1 State Key Laboratory of Natural and Biomimetic Drugs, School of Pharmaceutical Sciences; 2 Department of Chemical Biology, Peking University, Beijing 100191, China

## Abstract

The precise engineering of proteins in bacteria via the amber codon has been hampered by the poor incorporation of unnatural amino acid (UAA). Here we explored the amber assignment as a sense codon for UAA by CRISPRi targeting release factor 1 (RF1). Scanning of RF1 gene with sgRNAs identified target loci that differentiate RF1 repressions. Quantitation of RF1 repressions versus UAA incorporation indicated an increasing interrelation with the amber reassignment maximized upon RF1 knockdown to ~30%, disclosing the beneficial role of RF1 in amber assignment. However, further RF1 repression reversed this trend resulting from the detrimental effects on host cell growth, disclosing the harmful aspect of RF1 in reassignment of the amber codon. Our data indicate RF1 as a switch manipulating genetic code expansion and pave a direction via CRISPRi for precise engineering and efficient production of proteins in bacteria.

The UAG (amber) stop codon has been exploited for precise engineering of proteins in live cells^1^,^2^,^3^. With the aid of orthogonal tRNA and aminoacyl tRNA synthetase pairs, more than 100 unnatural amino acids (UAAs) with varying functional groups and side chains have been site-specifically incorporated into proteins in bacteria. However, a major limitation for amber-guided UAA incorporation is its low efficiency, which is possibly caused by release factor 1 (RF1) (Fig. 1A)^4^,^5^,^6^. RF1 possesses a conformation mimicking that of tRNAs and, thus, is able to compete with the suppressor tRNAs for the amber codon^7^. It remains to be elucidated that the role of RF1 in assignment of amber codon as either a sense codon, recognized by suppressor tRNA, or stop codon, recognized by RF1, or both in genetic code expansion^4^. The ambiguous assignment of the amber codon reduces the efficiency of UAA incorporation and makes the production of truncated proteins inevitable and even dominant in many circumstances. Furthermore, the low efficiency of UAA incorporation prevents the multiple UAA incorporation, limiting the potential and scope of genetic code expansion.

To address these issues, a series of pioneering work was performed to reassign the amber codon from being a stop signal into a sense codon^4^,^5^,^8^,^9^,^10^,^11^,^12^,^13^. Wang *et al*. demonstrated that RF1 can be knocked out in *Escherichia coli* upon fixing the expression of RF2, an alternative release factor that recognizes the stop codons UAA and UGA in prokaryotes^4^. Mukai *et al*. also successfully knocked out RF1 from the bacterial genome after externally supplying of seven essential genes with changing their stop codon from UAG to other stop codon^5^. An *E. coli* strain was even generated, in which 95 of the 273 TAG stop codons in its genome were replaced by exploiting the oligonucleotide-mediated base-mismatch-repair mechanism^13^. The resultant strains efficiently incorporated UAA at multiple UAG sites and tolerated global suppression of endogenous amber codons. In addition, Lajoie *et al*. generated a strain termed genomically recoded organism (GRO), in which all known UAG codons had been replaced by synonymous UAA codons. This GRO strain tolerated the deletion of RF1 for UAA incorporation^12^.

Despite of the significant achievement on reassignment of UAG as a sense codon, generation of RF1 knockout strains is usually time-consuming and prevents the application of genetic code expansion. In this study, we explored the amber assignment by CRISPR interference (CRISPRi) with RF1 gene being scanned by sgRNAs^14^,^15^. A variety of RF1 repressions were achieved with the incorporation of UAA enhanced but the cell growth lost. Quantitation of RF1 repression versus UAA incorporation and cell growth revealed RF1 as a double-edged sword mediating both beneficial and harmful effects on amber reassignment. The yields for incorporation of one to three UAAs were ~90%, ~50% and ~25%, respectively, upon optimization of RF1 knockdown. In addition, the CRISPRi cassette was engineered to be compatible with common vectors for applicable to different proteins and bacteria^16^. Thus, the combination of CRISPRi with genetic code expansion via a routine bacterial transformation established a facile and straightforward approach for precise engineering and high production of proteins of interest in bacteria.

## Results

### Scanning of the RF1 gene by CRISPRi for achieving RF1 repression

**Figure 1:**
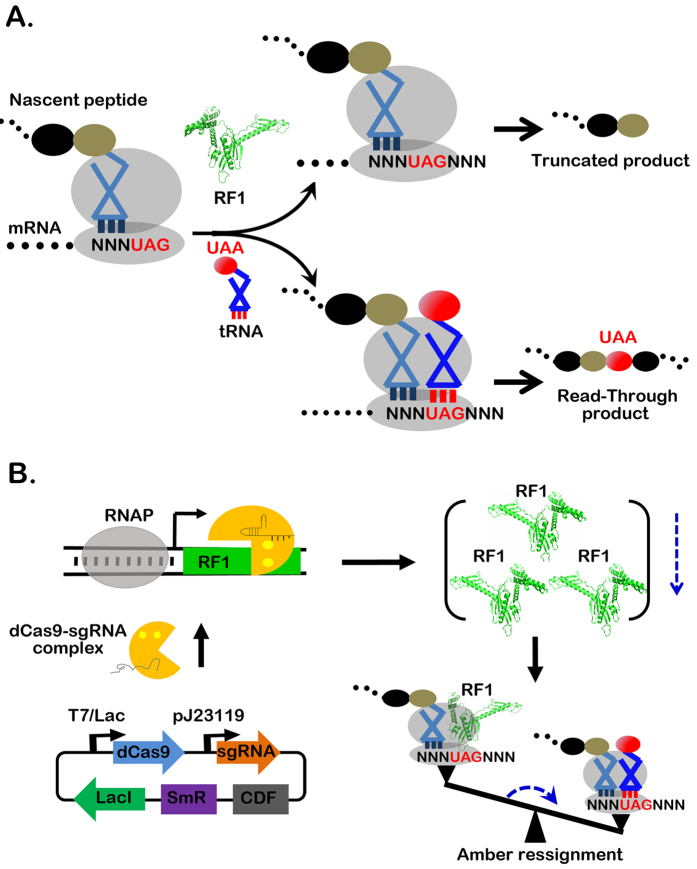
Schematic representative of assignment of amber codon as either a stop or encoding signal in genetic code expansion. (**A**) Schematic representative of the different translation routes resulting from the competition between RF1 and suppressor tRNA with the amber codon that leads to the truncated and read-through proteins as the respective products. (**B**) Schematic representative of the sgRNA-guiding, CRISPRi-mediated repression of RF1 for reassignment of amber from a stop codon to a sense codon.

**Figure 2:**
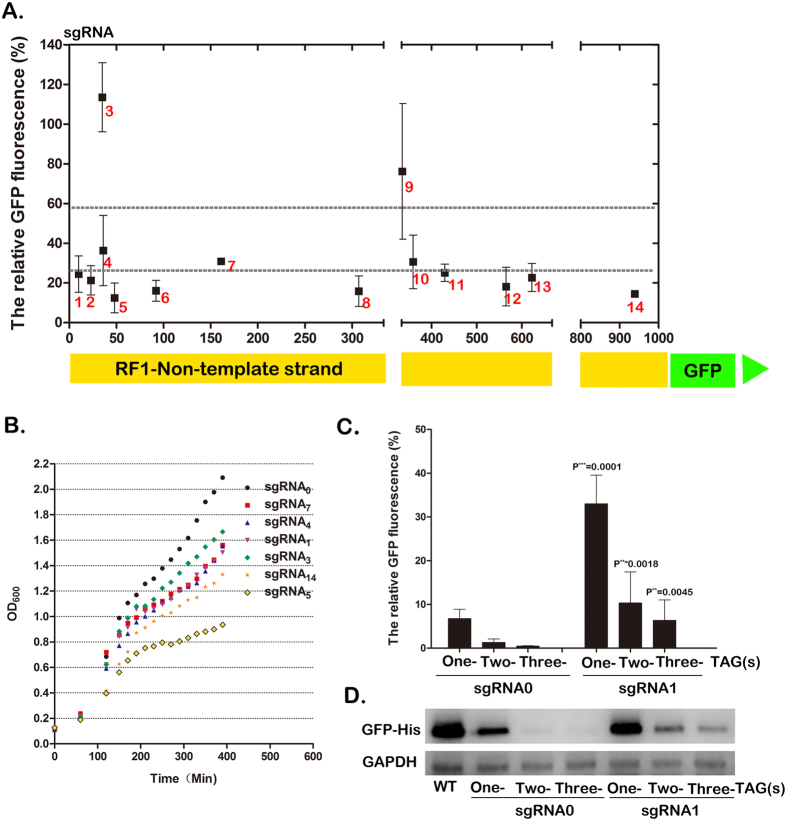
CRISPRi-mediated RF1 repression and the resulting effects on host cell growth and UAA incorporation into GFP protein. (**A**) Comparison of the knockdown effect of 14 sgRNAs designed complementary to the different loci of RF1 gene, which was fused with GFP as a reporter. The expression level of RF1 was quantitated by GFP fluorescence (485 and 520 nm) and normalized to the control sgRNA. Error bars show one standard deviation from the mean of at least three values. (**B**) The growth curves of BL21 (DE3) host strains transformed with pCCR-sgRNAx. The transformed strains were separately inoculated into Luria-Bertani (LB) medium containing 35 ug/ml spectinomycin and then grew overnight at 220 rpm under 37 ^o^C. Then the cultures were diluted to 0.1 with IPTG added to a final concentration of 0.5 mM. The OD600 values were measured every 20 minutes. (**C**) RF1 repression-induced incorporation enhancement of UAA into GFP gene containing one-, two- and three-TAG codons in BL21(DE3) strains. The GFP fluorescence per OD600 was measured relative to the wild-type GFP (WT) and was measured in 3 independent batches of cells. (**D**) Western blot analysis of the expression levels of GFP reporter, which contain one, two or three UAAs, upon knockdown of RF1 by sgRNA0 and sgRNA1, respectively, with GAPDH acted as an internal control.

Given the time-consuming approach for RF1 knockout in the pioneering work, we determined whether RF1 can be knocked down by CRISPRi in bacteria. We constructed plasmid pCCR-sgRNA_X_ which contains the expression cassettes of dCas9 and sgRNA_X_, two activator components of CRISPRi under the T7/lac and pJ23119 promoters, respectively (Fig. 1B). The later one is a synthetic and constitutive promoter amplified from pgRNA-bacterial for expression of sgRNA^14^. A rare CloDF13 replication (CDF) and spectinomycin antibiotic selectable marker (SmR) were also integrated to enhance the compatibility of CRISPRi with common vectors^16^. Fourteen sgRNAs, sgRNA1-14, were designed with their sequences spanning of the open reading frame of RF1 (Fig. 2A and S Table 1).

In order to exclude effect other than sgRNAs on RF1 repression such as cell growth, we constructed a reporter plasmid, pET21-RF1-GFP, which contains the fusion of RF1 with GFP for quantitative analysis (Fig. 2A). The reporter plasmid was co-transformed with each of the pCCR-sgRNA in parallel to *E. coli* BL21(DE3), a common strain used to produce a variety of proteins. After overnight incubation with 0.5 mM IPTG at 30 °C, the expression levels of RF1 in the transformants from single clones were measured and normalized to the control sgRNA0 (pCCR-sgRNA0), which contained no complementary sequence of the RF1 gene. We found that varying degrees of RF1 repression were obtained depending on the targeting sites of the sgRNAs (Fig. 2A). Significant repression (>80%) was observed for sgRNA5, sgRNA6, sgRNA8, sgRNA12, and sgRNA14; moderate repression (60–80%) was observed for sgRNA1, sgRNA2, sgRNA4, sgRNA7, sgRNA10, sgRNA11, and sgRNA13; and a slight to no effect was detected for sgRNA3 and sgRNA9. These results suggested that the RF1 gene can be knocked down by CRISPRi in bacteria, and the degree of repression is dependent on the complementary locus of sgRNA.

### Effect of CRISPRi-mediated RF1 repression on host cell growth

We then investigated whether knockdown of the endogenous RF1 causes any detrimental effects on cell growth. The BL21(DE3) strain was transformed in parallel with seven representative pCCR-sgRNAs, which repressed RF1 in varying degrees in the reporter assay. Single transformed colonies grown in LB media were selected, and their optical density (OD600) was measured at log phase growth. Compared with the strains transformed with the control pCCR-sgRNA0 (doubling time: ~45 min), the strains transformed with pCCR-sgRNA1, -sgRNA3, -sgRNA4, -sgRNA7, and –sgRNA14 exhibited a substantially reduced growth rate with a doubling time ranging from 60 to 70 minutes (Fig. 2B). The growth inhibition for each clone was correlated with the degree of endogenous RF1 repression as detected by real-time RT-PCR (S Fig. 1). In particular, the growth of the clone harboring pCCR-sgRNA5, which attenuated RF1 expression to a nearly undetectable level, was significantly reduced with an extended doubling time of up to 110 min. These results suggested that bacterial strains are generally tolerant to moderate but not complete RF1 knockdown. This was unsurprising because RF1-guided termination of protein translation is an evolved event in central dogma, accounting for 10% of the termination of endogenous protein translation in bacteria^17^. This finding was also consistent with previous observations that RF1 knockout led to deleterious defects on cell growth unless RF2 expression was fixed or additional genes were introduced.

### Effect of CRISPRi-mediated RF1 repression on incorporation of UAA

We then determined whether RF1 knockdown enhances the efficiency of UAA incorporation. pSURAR-YAS (a reported plasmid harboring the orthogonal pyrrolysyl tRNA-synthetase/tRNA_CUA_ pair used to incorporate DiZPK, a photo-crossing UAA^18^) and pET21-GFP_TAG_ (a GFP reporter plasmid containing a TAG codon at the Y39 position) were co-transformed with pCCR-sgRNA1 into *E. coli* BL21(DE3). Parallel transformations of the wild-type pET21-GFP and pCCR-sgRNA_0_ into BL21(DE3) were performed to act as the positive and negative controls, respectively. We found that the expression level of the mutant GFP, as revealed by GFP fluorescence, was ~30% that of the wild-type GFP but nearly six-fold higher than that of the control pCCR-sgRNA0 (Fig. 2C). We then investigated the expression of GFP containing two and three TAGs, which amber stop codon was introduced to replace the triplet codes for Y39/K101 for GFP(TAG_2_) and Y39/K101/E132 for GFP(TAG_3_), via reciprocal transformation of pET21-GFP (TAG_2_) and pET21-GFP (TAG_3_). As shown in Fig. 2C, significant GFP fluorescence was detected in both transformants, and the fluorescence intensity was nearly 10-fold higher than that of the negative control. This result was confirmed by Western blot analysis, which directly indicated the expression levels of GFP protein (Fig. 2D). By contrast, no apparent full-length GFP fusion protein was detected from the negative control. These results demonstrated that RF1 knockdown significantly improved the efficiency of the incorporation of either single or multiple UAAs. However, we also found that growth of the pCCR-sgRNA1 transformant was much slower than that of the control pCCR-sgRNA0, which was caused by the detrimental effect of RF1 knockdown.

### Evaluation of RF1 as a double-edged sword in reassignment of amber code

**Figure 3:**
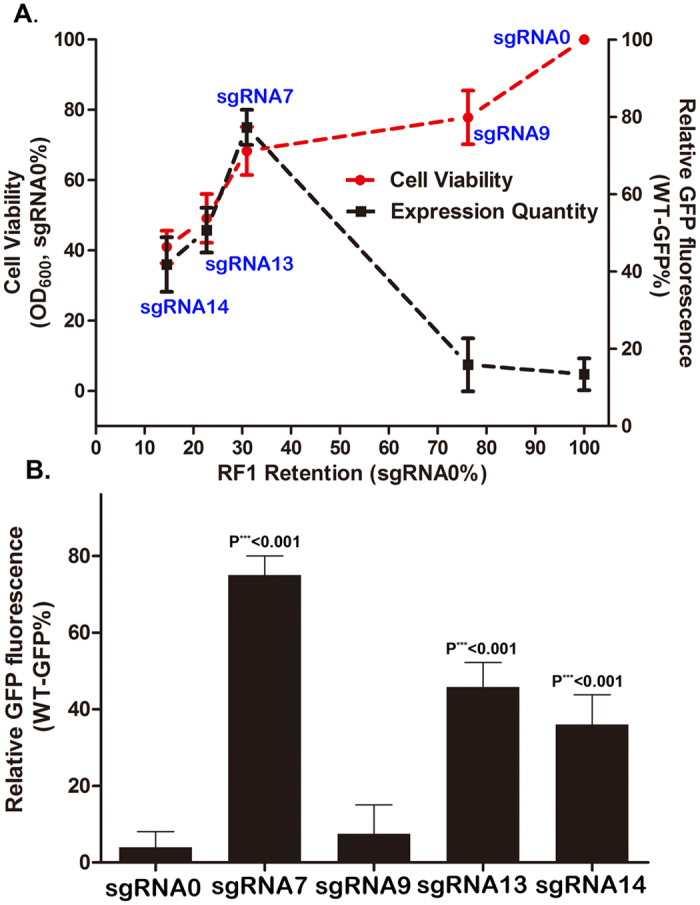
Evaluation of RF1 as a double-edged sword in reassignment of amber code. (**A**) Quantitation of RF1 repressions versus UAA incorporation and host cell growth detected by GFP fluorescence and OD600, respectively, when Y39NAEK-GFP was expressed. The values were measured relative to sgRNA_0_ and performed by three independent batches of cells. Error bars show one standard deviation from the mean of at least three values. (**B**) In-cell fluorescence assay of GFP signals in BL21(DE3) strains transformed with the representative pCCR-sgRNA plasmids that led to different extent of RF1 repression. The GFP fluorescence was measured relative to wild-type GFP (WT) value. P*** < 0.001 was determined by One-way ANOVA with Dunnett’s test to compare the expression levels in all experimental groups.

Upon verifying the feasibility of CRISPRi-mediated RF1-knockdown in *E. coli*, we elucidated the role of RF1 in reassignment of amber as a sense codon. pET21-GFP(TAG_1_) was co-transformed into BL21 (DE3) with pCCR-sgRNA which carries the representative sgRNA isomers across RF1. We monitored GFP fluorescence and cell viability (OD600) of the transformed cells. The results of the quantitation of RF1 repressions versus GFP fluorescence and cell growth indicated an increasing trend of UAA incorporation following the initial RF1 repression, as shown in sgRNA7 versus sgRNA9 versus sgRNA0 (Fig. 3A); a maximum of 20-fold improvement in UAA incorporation was obtained (Fig. 3B). In a reciprocal experiment testing cell growth, a slightly decreasing trend of cell viability was observed with continuing RF1 repression. However, the increasing trend was reversed upon RF1 knockdown crossing 30% because further RF1 repression reduced GFP fluorescence in the comparison of sgRNA7 versus sgRNA13 versus sgRNA14. The sgRNA13 and sgRNA14 exerted only ~12- and 9-fold improvement, respectively, although their effect on RF1 repression was much significant than that of sgRNA7 (Fig. 3B). This observation was due to the detrimental effects of RF1 knockdown on the host cell. Thus, RF1 was a double-edged sword mediating both beneficial and harmful effects on genetic code expansion. Knockdown of endogenous RF1 to ~30% by sgRNA7 was the turning point by which the reassignment of amber as a sense codon was maximized, whereas the detrimental effect on cell growth was minimized (Fig. 3B).

### Versatility of CRISPRi-mediated RF1 repression for genetic code expansion

**Figure 4:**
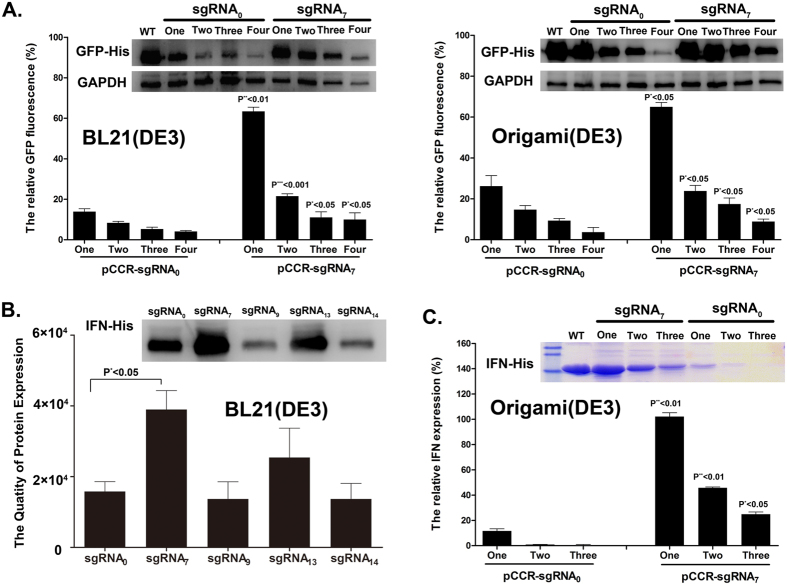
Test of the versatility of CRISPRi-mediated RF1 repression for genetic code expansion. (**A**) Comparisons of the effect of CRISPRi-mediated RF1 repression on GFP production in BL21(DE3) vs. OrigamiB(DE3) cells. The expression levels of GFP which contains from one to four UAAs were detected by both Western blotting and fluorescence. The later data were then normalized to that of the wild type GFP. Three independent batches of cells were measured. The Student t-Test was used to determine the statistical difference between means of group and all the P values are less than 0.05; (**B**) Comparisons of the effect of CRISPRi-mediated RF1 repression on quantitation of M111NAEK-IFN expression via Western blotting analysis. One-way ANOVA with Dunnett’s test was performedo the compare the expression difference all the experimental groups; (**C**) The sgRNA7-dependent production of IFN-α2 which contains from one to three UAAs analyzed by ComassieBlue staining and quantitated by each band normalized to WT-IFN-α2. IFN-α2 was purified with Ni-NTA chromatography and the loading was normalized to the equal volume of culture medium. The Student t-Test has been used to determine the statistical difference between means of group and all the P values are less than 0.05.

We then tested whether CRISPRi-mediated RF1 reassignment is applicable to other UAAs, proteins, and bacteria. Parallel experiments were performed, in which one of these components was changed in each experiment. In one parallel experiment, the UAA was replaced with Nε-2–azideoethyloxycarbonyl-L-lysine (NAEK), a handle amino acid for click-mediated conjugation of various chemical moieties^19^,^20^. We found that much higher GFP fluorescence and protein production (four- to six fold increase) were detected in strains containing sgRNA7 than in those containing sgRNA0 when either a single or multiple UAAs was incorporated into the GFP protein (Fig. 4). The GFP fluorescence and Western blot data of a reciprocal experiment showed a similar trend when the BL21(DE3) strain was replaced with Origami(DE3), a common *E. coli* strain exhibiting double deletion of *trxB* and *gor*, which promote disulfide formation. This result demonstrated that CRISPRi-mediated RF1 repression significantly enhanced the efficiency of UAA incorporation, particularly at optimized RF1 repression (Fig. 4A).

We then replaced the GFP reporter by IFN-α2, a therapeutic interferon for cancer and infectious diseases^21^. For IFN(TAG1), IFN(TAG2) and IFN(TAG3) proteins, the triplet codes for M111, M111/H34 and M111/H34/A74 were respectively mutated into the amber stop codon. We found that the yields of mutant IFN-α2 proteins were significantly enhanced, which correlated well with the extent of RF1 repression and cell viability (Fig. 4B and S Fig. 2). Maximum production of IFN-α2 was observed for sgRNA7 as compared with other sgRNAs. In particular, when the host bacteria were replaced with Origami(DE3), a further significant improvement in UAA incorporation was achieved as revealed by the quantitation of the IFN-α2 product using Coomassie Blue staining. The overall yields of the incorporation of one, two, and three UAAs at the turning point were ~90%, ~50%, and ~25%, respectively (Fig. 4C). This is significantly contrast to the control sgRNA0 in which much lower yields, 10%, of mutant IFN-α2 were produced when one TAG was introduced, and marginal IFN-α2, 1~2%, was detected for introduction of two TAGs and no IFN-α2 product was detected for introduction of three TAGs. The fidelity of UAA incorporation of GFP and IFN at the respective sites were verified by Mass spectrometry (S Fig. 3 and S Fig. 4). Thus, CRISPRi-mediated genetic code expansion has a significant effect on the precise engineering and high production of proteins of interest in bacteria.

## Discussion

CRISPRi is an approach complementary to RNAi for site-specific knockdown of genes of interest in prokaryotic cells^14^. The level of gene repression is dependent on the target loci which forms base pairing with sgRNA, the guiding component of CRISPRi. We found that different RF1 repressions were achieved via selection of the targeting sites. The accomplishment of CRISPRi-mediated RF1 repression via a routine bacterial transformation disclosed the fate of the amber codon as either a sense or stop codon, which is dependent on the competing binding of amber codon with either suppressor tRNA or endogenous RF1. This lead to different translation events with a read-through and truncated protein as the respective product (Fig. 1A).

Quantitation of the resultant RF1 repressions versus UAA incorporation and host cell growth disclosed that RF1 was a double-edged sword mediating both beneficial and harmful effects on genetic code expansion (Fig. 3A). The observed detrimental effect on cell growth was clearly due to RF1 knockdown in the host cell since RF1 plays the key role in protein translation termination. It is thus anticipated that any interference towards RF1 will inevitable affect the expression of endogenous genes with UAG as a termination signal. To clarify the consequence of RF1 repression, we detected the decay pathway mediated by tmRNA system^22^,^23^, which has been well known for translation of mRNAs that lack of in-frame stop codons (S Fig.5A). It was found that RF1 repression significantly activated the decay pathway with many endogenous proteins being added a tag and degraded lately (S Fig.5B), leading to the observed viability decrease (S Fig.5C).

In particular, repression of RF1 to ~30% was the threshold in which the reassignment of amber as a sense codon was maximized, whereas the detrimental effects on host cell growth were minimized. Under such an optimized condition the overall yields of incorporating one, two, and three UAAs were ~90%, ~50%, and ~25%, respectively. This not only confirms the prevail role of RF1 in amber assignment (Fig. 1B) but, more importantly, addresses the major issue limiting the potential and scope of genetic code expansion for different UAAs, proteins and bacteria hosts.

In conclusion, RF1 is proved as a potential switch in controlling the amber reassignment from as a stop signal to a sense codon. The combination of CRISPRi with genetic code expansion via a routine bacterial transformation elucidated the role of RF1 in reassignment of amber codon. It also established a versatile platform for precise engineering and efficient production of proteins in bacteria.

## Materials and Methods

### General materials

The unnatural amino acids Nε-2-azidoethyloxycarbonyl-L-lysine (NAEK) and ((3-(3-methyl-3H-diazirin-3-yl)propamino)carbonyl)-Nε-L-lysine (DiZPK) were synthesized as previously reported^18^,^24^. QuickChange Lightning Site-Directed mutagenesis kit (*Agilent Inc.*) was used to generate site-directed mutations. Phusion hot start Flex 2 × master mix (*New England Biolabs, Inc.*) and PCR clean-up System (*Promega Inc.*) were used to perform PCR and DNA fragment purification.

### Plasmid constructions

Plasmid pCCR-sgRNA was constructed by an In-fusion method. Briefly, the fragment of dCas9 was amplified from pdCas9-bacteria (*Addgene,* No. 44249) using the primer In-Fusion-F (5′-GGAGATATACCATGGATAAGAAATA CTCAATAGGCTTAGC-3′) and In-Fusion-R (5′-TCCCAATTGGGATCCATAAAA CGAAAGGCCCAGTCTTTCG-3′). The PCR product was cloned into vector pCDF-1b as manufacturer described (*TAKARA Inc*.). The gene encoding sgRNA was amplified from pgRNA-bacteria (*Addgene,* No. 44251) and cloned into the above constructed vector via PstI and PacI for generation of pCCR-sgRNA_0_. Construction of other pCCR-sgRNA plasmids with various sgRNA-coding sequences was carried out as previously reported^25^.

### Expression of IFN-2b-His-tag proteins

pET-IFN-alpha2b, a previously reported plasmid^22^, was transformed into BL21(DE3) or Origami(DE3) strains alone with pSURAR-YAV and pCCR-sgRNA plasmids. The transformed strains were inoculated into 2 ml LB medium containing 100 ug/ml ampicillin, 34 ug/ml chloramphenicol and 35 ug/ml streptomycin, and then cultured at 220 rpm under 37 °C. Then the overnight cultures were diluted to an optical density in 2 × YT medium and continuously incubated at 37 °C. When optical densities of the cultures reached ~0.5 (OD600), UAA was added to a final concentration of 1 mM. Then protein expression was induced by adding IPTG and L-Arabinose at a final concentration of 0.5 mM and 0.1%, respectively, half hour later. After overnight induction at 24 °C, cells were harvested by centrifugation and re-suspended in His-Bind buffer (20 mM phosphate, pH 8.0, 500 mM NaCl, 20 mM imidazole). Protein extraction was performed by passing cells through a Micofluidizer two times at 1200 bar with cooling. The supernatant was collected by centrifugation and the pellets were re-suspended in PBS buffer. The samples were analyzed on 15% SDS-PAGE gel followed by CoomassieBlue staining under reducing conditions.

### SDS-PAGE and Western Blot analyses

The protein concentration for Western blot analysis was determined by BCA method (*Thermo scientific*). 12% and 15% SDS-PAGE gels were separately used for analysis of GFP-His-tag and IFN-2b protein, respectively. For Western blot analysis of GFP-His-tag and IFN-2b-His-tag proteins, mouse monoclonal antibody anti-His6-tag (*TransGen Biotech*, HT501) diluted in 1:3000 was utilized a probe. GAPDH, as an internal loading control, was probed by anti-GAPDH antibody (*Santa Cruze*, sc-25778) in a 1:500 dilution. Then the above blots were probed with corresponding HRP-linked antibodies as secondary antibodies and detected with Immobilon^TM^ western HRP substrate (*Millipore*).

### RT-PCR and real-time PCR analyses

The reverse transcription of the total RNA and the real-time RT-PCR were conducted according to the protocol provided by *Promega Inc*. The transcripts were quantitated and normalized to the internal GAPDH control. The respective up- and down-primers utilized for RT-PCR analyses were 5′- AGTTGGTGGTGCAGGAAGCGTT-3′/ 5′- AACACATCACCGCTGGTGC GAA-3′ for GAPDH, and 5′- AACCGTACTTACAACTTCCCACA-3′/ 5′-AATCAG CATATCCAGCTTACCTTC-3′ for RF1 (919–1023bp of RF1 gene).

### Mass spectrometry analysis

The site-specific UAA incorporation was confirmed by peptide mapping. Following trypsin digestion of the purified WT-IFN-alpha2b (as control), H34/A74/M111NAEK-IFN-alpha2b (IFN-2b bearing UAA at site 34, 74 and 111) at 37 °C overnight, the digestion reaction was quenched by adding TFA. The tryptic peptides were re-suspended and separated by C18 RP-HPLC (Easy-nLC II, *Thermo Fisher Scientific, CA*) with in-line UV detection. Fractions were collected, dried down, and re-suspended in trifluoroacetic acid subsequently. Each fraction was separated with an analytical C18 column further. The peptide(s) were identified by MALDI (MS and MS-MS) analysis.

### Statistical analysis

Student’s t-test or one-way ANOVA with Dunnett’s test was utilized to analyze the data with p values < 0.05 being considered significantly.

## Additional Information

**How to cite this article**: Zhang, B. *et al*. CRISPRi-Manipulation of Genetic Code Expansion via RF1 for Reassignment of Amber Codon in Bacteria. *Sci. Rep.* **6**, 20000; doi: 10.1038/srep20000 (2016).

## Supplementary Material

Supplementary Information
