# Supplementary material for: CRISPRi-Manipulation of Genetic Code Expansion via RF1 for Reassignment of Amber Codon in Bacteria
Source: Sci Rep. 2016 Jan 28;6:20000. doi: 10.1038/srep20000 (PMC4730227; doi:10.1038/srep20000)
Supplement: Supplementary Information [file srep20000-s1.doc]

**Supporting Information**

CRISPRi-Manipulation of Genetic Code Expansion via RF1 for Reassignment of Amber Codon in Bacteria

Bo Zhang^1^‡, Qi Yang^2^‡, Jingxian Chen^1^, Ling Wu^1^, Tianzhuo Yao^1^, Yiming Wu^1^, Huan Xu^1^, Lihe Zhang^1^, Qing Xia^2^*, Demin Zhou^1^*

^1^State Key Laboratory of Natural and Biomimetic Drugs, School of Pharmaceutical Sciences, ^2^Department of Chemical Biology, Peking University, Beijing 100191, China;

**S Table 1. The RF1-targeting sgRNA used in report**

|  | | **SEQUENCE*** | |
| --- | --- | --- | --- |
| sgRNA-0 | --- **N _(handle and terminator region)_** | |  |
| sgRNA-1 | TCCAGTTTGGCAACGATAGA**N _(handle and terminator region)_** | |  |
| sgRNA-2 | TTCATGCAGGGCTTCCAGTT**N _(handle and terminator region)_** | |  |
| sgRNA-3 | TTCTTCATGGCGTTCATGCA**N _(handle and terminator region)_** | |  |
| sgRNA-4 | CTTCTTCATGGCGTTCATGC**N _(handle and terminator region)_** | |  |
| sgRNA-5 | GCAACGCCTGAACTTCTTCA**N _(handle and terminator region)_** | |  |
| sgRNA-6 | TGCGCGAAAACGTTCCTGGT**N _(handle and terminator region)_** | |  |
| sgRNA-7 | TTCCTGAACCTGTTGCCAGT**N _(handle and terminator region)_** | |  |
| sgRNA-8 | CGTTCGTCATCAGGATCTTT**N _(handle and terminator region)_** | |  |
| sgRNA-9 | TCCGGCACGGACTTCGAGGA**N _(handle and terminator region)_** | |  |
| sgRNA-10 | CAGCGCCGCTTCGTCGCCGC**N _(handle and terminator region)_** | |  |
| sgRNA-11 | TCATGATTTCTACCCGCCAG**N _(handle and terminator region)_** | |  |
| sgRNA-12 | CGCCCCTGCGATTCCGTAGC**N _(handle and terminator region)_** | |  |
| sgRNA-13 | AGTTCTGCGTCAGGCAGCTC**N _(handle and terminator region)_** | |  |
| sgRNA-14 | GATCGGTAACGCGCCCCTGT**N _(handle and terminator region)_** | |  |

*only the 20 matching region is shown

***S Figure 1.*** qRT-PCR analysis of RF1-Knockdown form E.coli genome. A monoclonal culture of E.coli was grown at 37℃ to early logphase (OD~0.5), at which point the cells were harvested by centrifugation. The total RNA was extracted and purified according to manual instruction (Promega). Error bars show one standard deviation from the mean of at least three values.


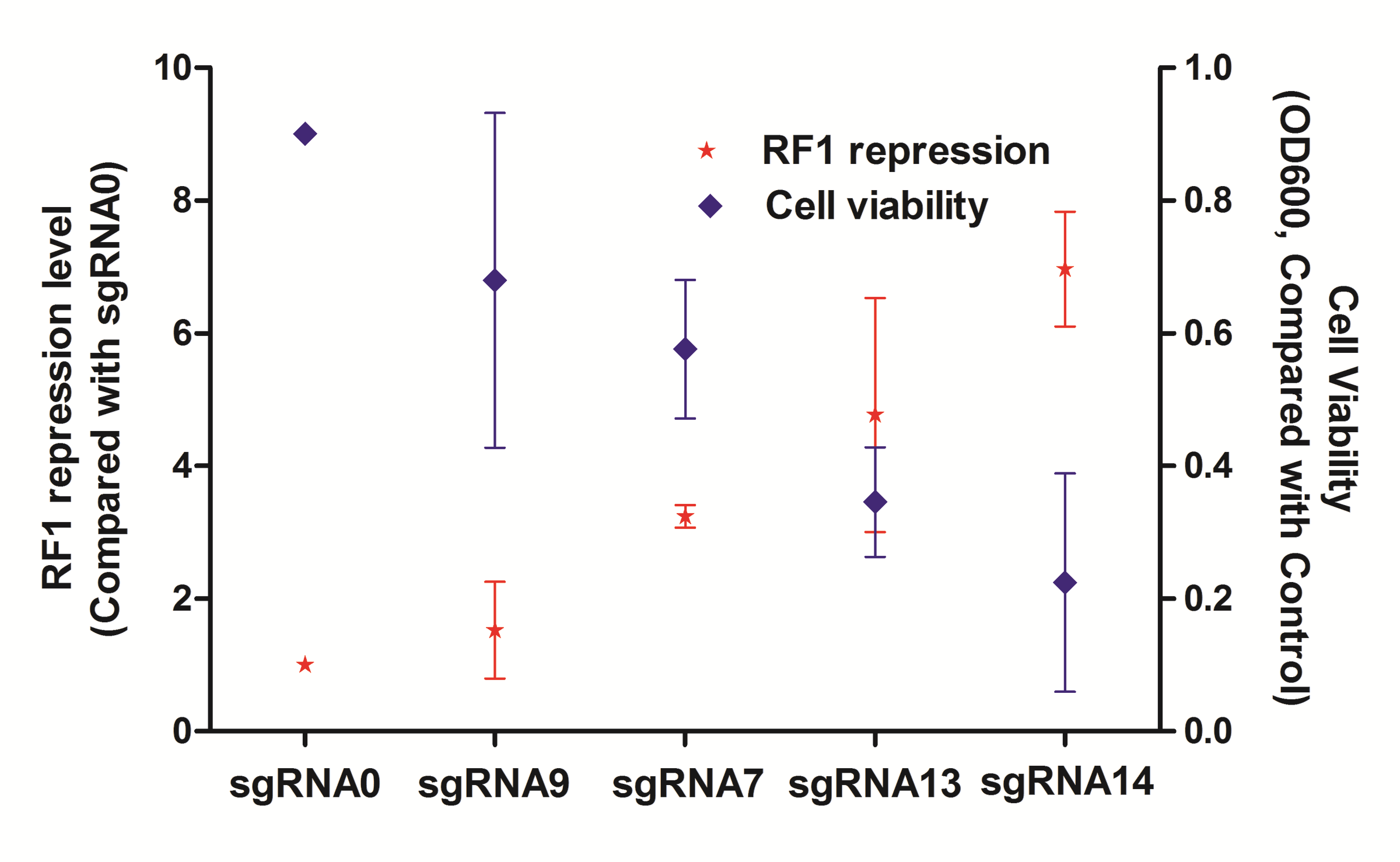


***S Figure 2.* Quantitation of RF1 repressions versus host cell growth detected by OD600 in expression of mutant IFN-α2 protein (M111NAEK)**. Only one TAG was introduced to replace the triplet code for M111 for demonstration of the relationship of RF1 repression level versus host cell viability. The repression extents of RF1 by different sgRNAs were measured and normalized to that of sgRNA0 which has been defined as 1. All experimental data were obtained from three independent batches of cells. Error bars showed one standard deviation from the mean of at least three data.


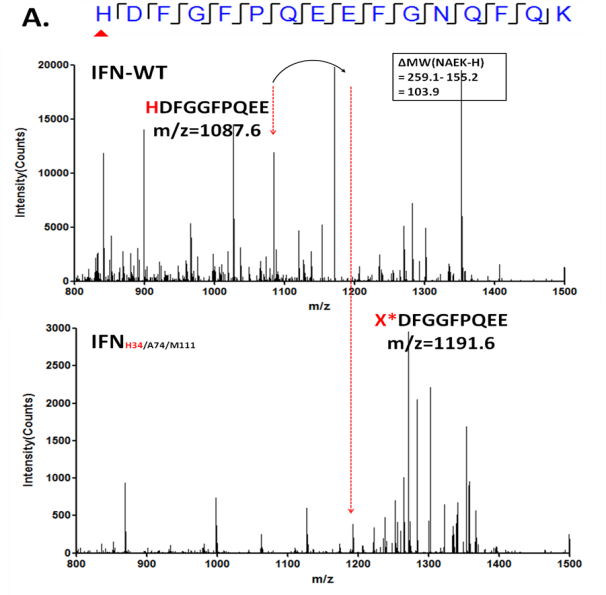

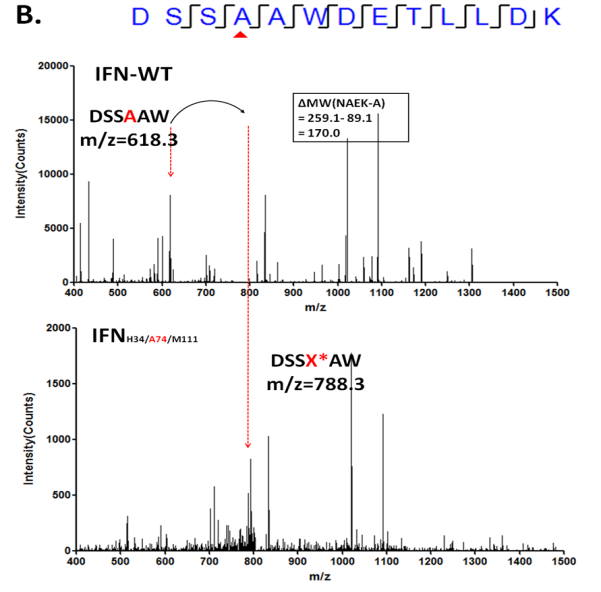


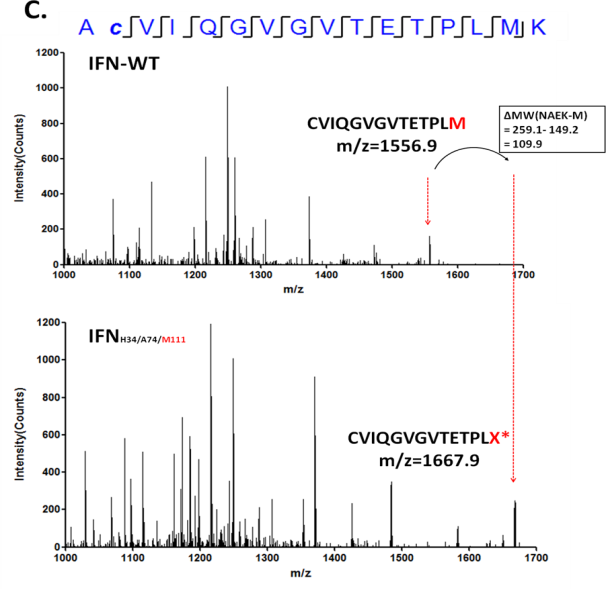


***S Figure 3.*** Peptide sequencing of IFN-a2b for validation of the incorporation of NAEK at the chosen site. The purified proteins were analyzed by SDS–PAGE and the corresponding bands were excised for MS analysis. X*denotes NAEK in IFNH34/A74/M111 or Histidine (H), Alanine (A) and Methionine (M) in WT-IFN at the position of 34, 74 and 111 respectively. m/z of peptides after tryptic digestion of IFN-WT at corresponding position were 1087.6, 618.3 and 1556.9 respectively; m/z of peptides after tryptic digestion of IFNH34/A74/M111 containing three NAEK at the corresponding position were 1191.6, 788.3 and 1667.9. The differences of corresponding peptides within IFN-WT and IFNH34/A74/M111 were equal to the value of NAEK minus H, A and M respectively. The margin of error is ±1.


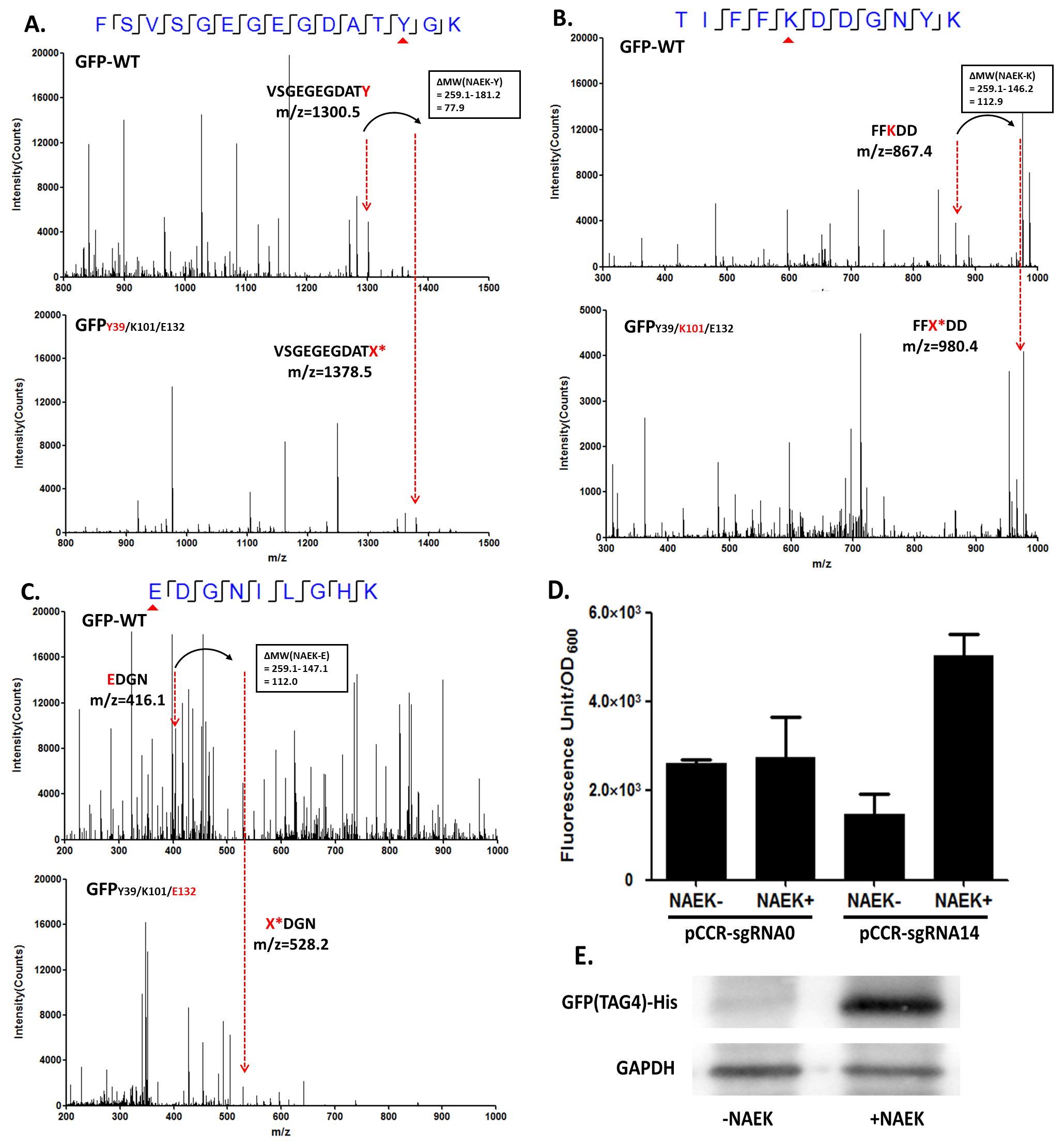


***S Figure 4.*** Peptide sequencing of GFP for validation of the incorporation of NAEK at the chosen site. The purified proteins were analyzed by SDS–PAGE and the corresponding bands were excised for MS analysis. X*denotes NAEK in GFPY39/K101/E132 or Tyrosine (Y), Lysine (K) and Glutamic acid (E) in WT-GFP at the position of (A) 39, (B) 101 and (C) 132 respectively. m/z of peptides after tryptic digestion of WT-GFP at corresponding position were 1300.5, 867.4 and 416.1 respectively; m/z of peptides after tryptic digestion of GFPY39/K101/E132 containing three NAEK at the corresponding position were 1378.5, 980.4 and 528.2. The differences of corresponding peptides within WT-GFP and GFPY39/K101/E132 were equal to the value of NAEK minus Y, K and E respectively. The margin of error is ±1. (D) Quantitation of GFP with four NAEK incorporation (GFP-Y34/K101/E132/E171, GFP(TAG4)) detected by GFP fluorescence. (E) Analysis of NAEK-engineered GFP(TAG4) expression in BL21(DE3) detected by Western Blotting. The full size of GFP could be only detected (The C-terminal His-tag blotting) in the presence of NAEK in the culture.

**
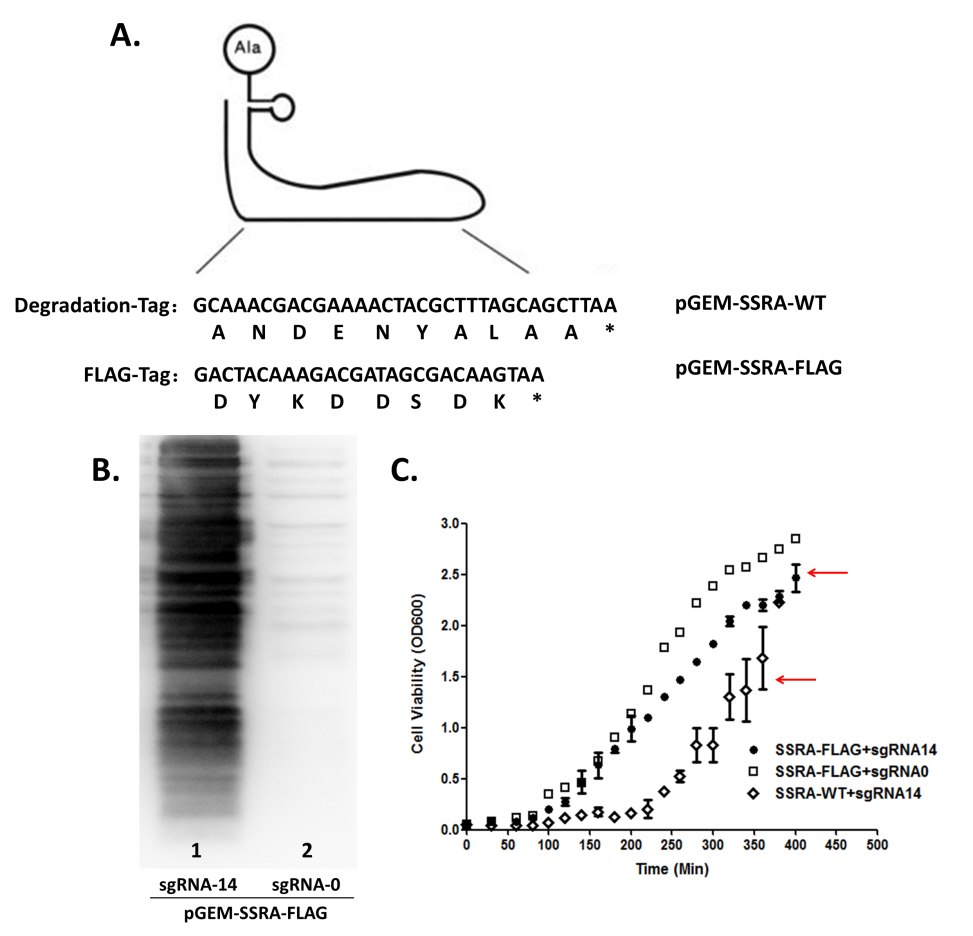
**

***S Figure 5.*** The degradation of endogenous proteins after RF1 repression mediated by tmRNA system. (A) Schematic drawing of SsrA-WT and SsrA-FLAG. The nucleotides in the tag-coding region of SsrA-WT, which are translated as a degradation-tag, are replaced with the sequences of FLAG-tag, a detected tag commonly used. The nucleotide and amino acid sequences of two genes are shown below the diagram. The gene of Ssra-WT and SsrA-FLAG was inserted into the pGEM-T vector and constructed as pGEM-SSRA-WT and pGEM-SSRA-FLAG, respectively. (B) Detection of tagging of cellular proteins mediated by RF1 repression. Total proteins equivalent to OD600=0.1, prepared from bacterial BL21(DE3) cells harboring pGEM-SSRA-FLAG and pCCR-sgRNA14 (Lane 1), pGEM-SSRA-FLAG and pCCR-sgRNA0 (Lane 2), were analyzed by western blotting using anti-FLAG-tag antibody. Compared with sgRNA0, our result demonstrated that many endogenous proteins are added tag and ready to be degraded lately. (C) The growth curves of BL21(DE3) host strains transformed with pGEM-SSRA-FLAG/pCCR-sgRNA14, pGEM-SSRA-FLAG/ pCCR-sgRNA0, pGEM-SSRA-WT/pCCR-sgRNA14, respectively. As arrow indicated, the viability of cells was significantly decreased under repression of RF1 and overexpression of SsrA-WT.
